# Breaking the Monologue With Active Learning Using the Think-Pair-Share Strategy: A Cross-Sectional Descriptive Study

**DOI:** 10.7759/cureus.95487

**Published:** 2025-10-27

**Authors:** Deepti Chopra, Yangshen Lhamo, Jaspreet K Sidhu, Sumit Kumar, Ashish Kumar

**Affiliations:** 1 Pharmacology, All India Institute of Medical Sciences, Bilaspur, Bilaspur, IND; 2 Pharmacology, Amrita School of Medicine, Amrita Vishwa Vidyapeetham, Faridabad, IND

**Keywords:** active learning, cooperative learning, critical thinking, medical education, think-pair-share strategy

## Abstract

Background

Modern educational methodologies increasingly focus on collaboration and active learning. Cooperative learning strategies, such as the Think-Pair-Share (TPS) strategy, foster active participation and critical thinking. This study aimed to assess the perceptions of second-year MBBS students regarding the effectiveness of the TPS strategy in learning pharmacology.

Methodology

The present study was conducted among 100 second-year MBBS students during a pharmacology lecture. The TPS activity was implemented by organizing students into “Think,” “Pair,” and “Share” phases. Feedback was obtained through a questionnaire. Quantitative data were analyzed using SPSS version 30 (IBM Corp., Armonk, NY, USA), while thematic analysis was performed for qualitative data.

Results

In total, 93 students provided feedback, with 86% (n = 80) reporting enhanced engagement and enjoyment with the TPS activity, 79% (n = 71) highlighting active participation, and 76% (n = 70) benefiting from increased comprehension of the subject content. Students found the strategy to be more effective than traditional lectures.

Conclusions

TPS proved to be an effective teaching strategy, increasing student engagement, comprehension, and participation during pharmacology lectures.

## Introduction

Modern educational methodologies emphasize collaboration, encouraging learners to discuss issues, solve problems, participate in simulations, and think critically. Cooperative learning strategies serve as instructional tools where students collaborate to optimize their own learning as well as that of their peers. Cooperative learning fosters positive interactions among students and promotes individual accountability. Learners acquire knowledge because they share experiences with peers [1]. It helps students analyze different perspectives and synthesize information better [2]. It also provides an opportunity for students to learn actively rather than just passively receive information [3,4].

The Think-Pair-Share (TPS) activity represents an innovative approach to cooperative learning that fosters interaction and active student participation within classrooms. By engaging in experiential learning, students improve their retention of information and acquisition of skills [5]. Students have more time to think, thus enhancing higher-order thinking. They become more involved in discussions, which improves the quality of their answers [6]. The combined effect of individual preparation and receiving validation of ideas from their peers has been shown to increase the self-confidence of students [6]. Despite its potential advantages, the implementation of TPS in health education remains largely unexplored. Pharmacology is a complex subject, and students often find it difficult to understand. The TPS activity is a structured method that can promote engagement and understanding [7]. Therefore, this study aimed to assess the perception of second-year medical undergraduate students toward the use of the TPS activity as a method to learn and understand pharmacology.

## Materials and methods

This study was conducted in accordance with the Declaration of Helsinki and involved anonymous responses of perceptions; therefore, formal ethical approval was deemed unnecessary. This cross-sectional study was conducted in July 2024 as part of a scheduled pharmacology lecture. The faculty facilitators were trained to conduct the TPS activity by the First author (DC), with more than 20 years of experience as a medical educator. One two-hour session was planned for the activity. All second-year MBBS students were included in the TPS session.

Intervention

The TPS strategy was employed to enhance active learning during a two-hour session on drugs modulating the renin-angiotensin-aldosterone system. For pre-lecture preparation, students received lecture material via the messaging app WhatsApp one week before the class. This helped students develop a preliminary understanding and enhance the quality of classroom discussion. The TPS activity was explained, emphasizing its role in encouraging discussion and idea sharing.

During the lecture, students first engaged in a “Think” phase; after that, they entered the “Pair” phase. Selected students then presented their discussions to the class during the “Share” phase. In the Think Phase (three minutes), a challenging question related to the management of a case was posed to the students. Students were given time to think individually about their answers and were instructed to complete the tasks on the provided worksheet to ensure individual participation and accountability. In the Pair phase (five minutes), following the thinking phase, students were paired with a nearby classmate to discuss their answers. The pairs were allotted five minutes to discuss. They were encouraged to listen attentively to each other and compare and elaborate on their thoughts. In the Share phase (5-10 minutes), one student from the pair was randomly selected or volunteered to share their thinking with the rest of the class. For Closure (10 minutes), feedback was given to reinforce key points and clarify misunderstandings. A brief summary of the main points was presented to reinforce important concepts.

The questionnaire was shared through Google Forms via the WhatsApp messaging application with all students. The questionnaire was developed by two authors (DC, YL). It was prepared after a review of the relevant literature. The questionnaire included seven questions, including both closed- and open-ended items to meet the study objectives. The open-ended questions concerned the usefulness, advantages, and disadvantages of the TPS activity. The validity was ensured by open-ended feedback from senior faculty members and subject experts not directly involved in the study. Feedback focused on the clarity, relevance, and comprehensiveness of each item and potential for bias in question phrasing. Based on these inputs, the questionnaire was revised further to improve its readability and minimize ambiguity and response bias. For closed-ended questions, responses were collected using a five-point Likert scale.

The participants were allowed to provide informed consent by completing the questionnaire. All responses collected were confidential and anonymous, with no personal information collected, ensuring participants’ privacy. The participants were given 15 minutes to respond to the questionnaire. Filling out the feedback questionnaire was purely voluntary. A total of 93 out of 98 students completed the questionnaire voluntarily, resulting in a response rate of 95%.

Statistical analysis

SPPS version 30 (IBM Corp., Armonk, NY, USA) was used to analyze the data. Descriptive statistical analysis tests were performed, and data were expressed as a percentage. Chi-square goodness-of-fit tests were done to evaluate the response perceptions toward the TPS strategy. Analysis of responses to the open-ended questions was conducted using thematic analysis. Thematic analysis of the open-ended responses was performed using an inductive approach. Two authors (DC, YL) independently reviewed all student responses, carefully reading and identifying relevant codes. These codes were compared, and consensus was achieved through iterative discussions. The agreed-upon codes were then organized into themes that represented the collective views expressed. The final thematic framework was reviewed and approved by all authors.

## Results

**Figure 1:**
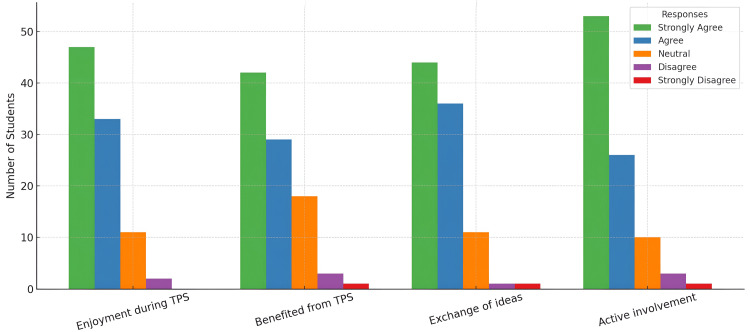
Students’ perceptions of the Think-Pair-Share (TPS) activity.

The overall perception responses were favorable, as shown in Figure 1.

Engagement and enjoyment

The vast majority of participants found that the TPS activity engaged them and created interest in the lecture. Overall, 86% (n = 80) of the students enjoyed the TPS strategy and found it to be fun in the classroom. Table 1 describes the students’ perception of the TPS activity.

**Table 1:** Students’ perceptions of the Think-Pair-Share activity.

Questions	Strongly disagree, n (%)	Disagree, n (%)	Neutral, n (%)	Agree, n (%)	Strongly agree, n (%)	Chi-square goodness-of-fit test
Do you find that there is fun and you enjoy applying the Think-Pair-Share strategy during the lecture?	0 (0)	2 (2)	11 (12)	33 (36)	47 (51)	Χ^2^ = 91.03; p < 0.001
Did you benefit from applying the Think-Pair-Share strategy during the lecture?	1 (1)	3 (3)	18 (19)	29 (31)	42 (46)	Χ^2^ = 65.01; p < 0.001
Do you think there is an exchange and sharing of opinions, thoughts, and ideas between you and your colleague when applying the Think-Pair-Share strategy during the lecture?	1 (1)	1 (1)	11 (12)	36 (39)	44 (47)	Χ^2^ = 87.38; p < 0.001
Did the Think-Pair-Share strategy in the lecture keep you actively involved in the discussion?	1 (1)	3 (3)	10 (11)	26 (28)	53 (57)	Χ^2^ = 100.28; p < 0.001

Active learning

Approximately 79% (n = 73) of students felt that the TPS activity made them actively participate in the class compared to the traditional didactic lectures.

Peer learning

Overall, 86% (n = 80) of the students were of the opinion that the TPS activity helped them interact with their peers and fostered meaningful discussion and sharing of ideas.

Understanding the topic

Further, 76% of students (n = 71) benefited conceptually from the TPS activity, which helped them increase their understanding of the topic. Only a small minority of students reported neutral or unfavorable responses (n = 11) (12%), further highlighting the largely positive perception among students for the TPS activity. Students were asked open-ended questions about the perceived usefulness and the advantages and disadvantages of the TPS activity, as illustrated in Table 2.

**Table 2:** Students’ responses to open-ended questions.

Question	Codes	Theme	Representative comments
The Think-Pair-Share strategy is useful	Active engagement in learning	Improved understanding and psychological safety	“Like putting pieces of a puzzle together”
	Reflection before discussion and collaborative learning		“Thinking individually before discussing with a peer allowed me to recognize and correct my mistake without the fear of embarrassment”
	Self-correction of errors and better understanding		“I can share more confidently after pairing”
What were the facilitating factors during the activity?	Maintained attentiveness and more discussion more learning	Student engagement and increased focus	“Teaching my friend improved my learning more”
What were the hindering factors during the activity?	Dependency on active participation and poor discussion with an inactive partner	Challenges of unequal participation	“If any one of the pair is inactive the quality of the discussion will be poor and the whole exercise will not be fruitful”

## Discussion

The TPS is an active learning strategy introduced by Professor Frank Lyman in 1981 [8]. Over the years, educators globally have adopted this method to enhance student interaction and learning experience [8-11]. This approach actively engages students by encouraging independent thought, collaborative discussions, and class-wide sharing. However, its application in medical education is still lacking. In this study, we analyzed the perception of students regarding the TPS activity as an instructional tool to teach pharmacology. This innovative method will shift the traditional teaching method to an active learning activity and promote deep learning instead of rote learning in medical education. Further, it is low-cost and easy to implement and can be adapted across various disciplines, from basic sciences to clinical case discussions.

The result of the present study showed that students believed that the TPS activity was more engaging than traditional teaching methods. They felt more involved in the lectures. These findings are consistent with other studies that showed that the TPS activity helps in active engagement of students in classrooms [9].

Various studies have also indicated that incorporating elements of fun and enjoyment into adult learning strategies motivates as well as increases the participation of students in class [12]. This perception of enjoyable learning plays a vital role in sustaining student interest, particularly in challenging subjects such as pharmacology. Similar findings were observed in our study, as most students (86%) found that the TPS activity made learning concepts of pharmacology enjoyable during the lecture.

The TPS activity begins with the teacher posing a question, giving students time for independent reflection. This critical first step promotes logical thinking and reasoning. Subsequently, students pair up with their peers to exchange and deliberate on their ideas. Tanner (2009) emphasized that this peer interaction encourages recalling and processing of the learned concepts [12]. Studies have shown that when students talk and share ideas in groups, they participate in activities such as observing, thinking, explaining, asking questions, and planning. These activities help them build and improve their critical thinking skills [13].

The pairing stage helps students actively connect with the content, making learning more interactive instead of passive. Group discussions allow them to improve their ideas, clear up any confusion, and strengthen their understanding without the fear of judgment. Explaining concepts to peers has been observed to facilitate better comprehension and retention. Research suggests that students often find explanations from classmates easier to follow than those given by teachers. This makes the TPS method a powerful way to encourage critical thinking and deepen understanding of complex subjects. This is in line with the results of our study, as most students were of the opinion that the TPS activity improved their understanding of the topic compared to the didactic lectures. The responses of the students suggested that the TPS strategy helped them interact with their peers and fostered meaningful discussion and sharing of ideas. One of the students stated, “Teaching my friend improved my learning more,” suggesting the same. In the final step, the teacher leads a discussion with the entire class, giving students a chance to share their ideas.

The majority of the students perceived that this method improved active class participation. This is consistent with the findings of Guenther and Abbott [13]. By letting students discuss and ask questions in pairs, this approach takes away the stress of speaking in front of the whole class [14].

Many studies have also shown that active participation in class leads to better academic performance over time [13-16]. However, the traditional teaching methods often struggle to get students actively involved because of issues such as shyness and self-consciousness. [13]. This may interfere with learning and in-class participation, leading to poor academic outcomes. The TPS activity overcomes these challenges by encouraging students to share ideas and discuss with their peers. It has been observed that TPS can improve the in-class participation of shy students. The combined effect of individual preparation and receiving validation of their ideas from their peers might increase the self-confidence of students and improve their learning outcomes [16].

Limitations

This study did not analyze the long-term impact on knowledge retention among students. Moreover, the participants were sampled from only second-year MBBS students in a college in Northern India, possibly affecting the generalizability of results. This single-center study relied on self-reported data, which may introduce response and faculty-student bias. Additionally, the absence of pre- and post-intervention assessments limits the objective evaluation of learning outcomes.

## Conclusions

The TPS activity enhanced student engagement and comprehension during the second-year MBBS pharmacology lecture. Student feedback indicated that TPS improved the quality of discussions and made the session more enjoyable. This approach fostered active participation and encouraged peer interaction throughout the lecture. It also enhanced deep learning instead of rote learning, which is prevalent in medical education. Given the positive outcomes, incorporating cooperative learning techniques such as TPS in the medical education curriculum can promote active learning and enhance students’ understanding of complex concepts. Further, TPS can be integrated with objective performance measures. As it requires very few resources, it can be implemented across multiple institutions.

## References

[1] Scager K, Boonstra J, Peeters T, Vulperhorst J, Wiegant F (2016). Collaborative learning in higher education: evoking positive interdependence. CBE Life Sci Educ.

[2] Johnson DW, Johnson R (2016). Cooperative learning and teaching citizenship in democracies. Int J Educ Res.

[3] Pateşan M, Balagiu A, Zechia D (2016). The benefits of cooperative learning. Int Conf Knowl-Based Org.

[4] Boedeker P, Schlingmann T, Kailin J (2025). Active versus passive learning in large-group sessions in medical school: a randomized cross-over trial investigating effects on learning and the feeling of learning. Med Sci Educ.

[5] Kong Y (2021). The role of experiential learning on students' motivation and classroom engagement. Front Psychol.

[6] Mundelsee L, Jurkowski S (2021). Think and pair before share: effects of collaboration on students' in-class participation. Learn Individ Differ.

[7] Fakoya AO, Ndrio M, McCarthy KJ (2023). Facilitating active collaborative learning in medical education; a literature review of peer instruction method. Adv Med Educ Pract.

[8] Cooper KM, Schinske JN, Tanner KD (2021). Reconsidering the share of a Think-Pair-Share: emerging limitations, alternatives, and opportunities for research. CBE Life Sci Educ.

[9] Silva H, Lopes J, Dominguez C, Morais E (2022). Think-pair-share and roundtable: two cooperative learning structures to enhance critical thinking skills of 4th graders. Int Electron J Elem Educ.

[10] Finn JD, Zimmer KS (2012). Student engagement: what is it? why does it matter?. Handbook of Research on Student Engagement.

[11] Crozier WR (2010). Shyness and the development of embarrassment and the self-conscious emotions. The Development of Shyness and Social Withdrawal.

[12] Tanner KD (2009). Talking to learn: why biology students should be talking in classrooms and how to make it happen. CBE Life Sci Educ.

[13] Guenther AR, Abbott CM (2024). Think-Pair-Share: promoting equitable participation and in-depth discussion. PRiMER.

[14] Márquez J, Lazcano L, Bada C, Arroyo-Barrigüete JL (2023). Class participation and feedback as enablers of student academic performance. SAGE Open.

[15] Aji CA, Khan MJ (2019). The impact of active learning on students' academic performance. Open J Soc Sci.

[16] Cortright RN, Collins HL, DiCarlo SE (2005). Peer instruction enhanced meaningful learning: ability to solve novel problems. Adv Physiol Educ.

